# Impact of Cardiovascular Diseases on Ischemic Stroke Outcomes

**DOI:** 10.31083/j.jin2105138

**Published:** 2022-07-26

**Authors:** Christa C. Huber, Xuejun Wang, Hongmin Wang

**Affiliations:** 1 Division of Basic Biomedical Sciences and Center for Brain and Behavior Research, Sanford School of Medicine, University of South Dakota, Vermillion, SD 57069, USA

**Keywords:** ischemic stroke, comorbidity, peripheral, heart, cardiovascular, protein aggregates

## Abstract

Stroke induces complex pathological cascades in the affected brain area, leading to brain injury and functional disability. To fight against cerebral ischemia/reperfusion-induced neuronal death, numerous neuroprotective strategies and reagents have been studied. However, translation of these neuroprotective drugs to clinical trials has been unsuccessful. To date, the tissue plasminogen activator is still the only FDA-approved drug for treating ischemic stroke. Thus, it is obligatory to identify and validate additional therapeutic strategies for stroke. A stroke rarely occurs without any other pathophysiological condition; but instead, it often has multi-morbidity conditions, one of which is cardiac disease. Indeed, up to half of the stroke cases are associated with cardiac and large artery diseases. As an adequate blood supply is essential for the brain to maintain its normal function, any pathophysiological alterations in the heart are frequently implicated in stroke outcomes. In this review, we summarize some of the cardiovascular factors that influence stroke outcomes and propose that considering these factors in designing stroke therapies should enhance success in clinical trials. We also highlight the recent advances regarding the potential effect of protein aggregates in a peripheral organ, such as in the heart, on ischemic stroke-caused brain injury and functional recovery. Including these and other comorbidity factors in the future therapeutic strategy designs should facilitate translational success toward developing effective combinational therapies for the disorder.

## 1. Introduction

Stroke is the fifth leading cause of death in the United States and results in severe long-term disability [1,2]. In the United States, the direct and indirect cost of stroke was $52 billion in 2017 [3]. By 2030, nearly 4% of the people in the United States over the age of 18 will likely suffer from a stroke [4]. With the increasing prevalence and the debilitating nature of the disease, it is imperative to understand more about the disease’s complex etiology.

A stroke occurs when blood flow to the brain is disrupted, causing not only physical disability but also cognitive impairment and epilepsy that affect the quality of life of stroke survivors [5]. Strokes can be classified as hemorrhagic or ischemic, the latter being the most common [6]. A hemorrhagic stroke happens because of a ruptured blood vessel that leads to bleeding in the brain, while an ischemic stroke occurs when an artery supplying blood to the brain region is blocked or greatly narrowed by a blood clot. Ischemic stroke creates a core area of irreversible damage surrounded by the penumbra, an area of the brain with reversibly injured tissue [7]. The decrease in oxygen and glucose supply to the ischemic area results in neuronal death. Restoring blood flow to the ischemic area is important; however, this has its own set of consequences, including reperfusion injury. As a result, therapeutic agents that limit cerebral ischemia/reperfusion-induced neuronal death are necessary to improve patient outcomes for stroke.

Currently, the only FDA-approved thrombolytic therapy for acute ischemic stroke is the tissue plasminogen activator, which must be given to a stroke patient in a timely manner. Even though this is an FDA-approved therapy for stroke patients, reperfusion therapies are not conducive for all patients. In addition, many neuroprotective agents tested in animal models yield promising results of improved neurological scores and reduced infarct size; however, translation of these neuroprotective agents has largely failed in clinical trials. The unsuccessful translation of these neuroprotective agents could be due to the multimorbid conditions present in patients who suffer from stroke, which are unable to be recapitulated in animal models. Only 6% of strokes occur without the presence of other conditions, suggesting multimorbid conditions play a key role in functional recovery for stroke patients [8]. The purpose of this review is to focus on cardiovascular factors that influence stroke outcomes, as up to 50% of strokes are associated with cardiac and large artery diseases. In addition, cardiovascular risk factors are seen in other stroke etiologies and influence a patient’s outcome [9]. Including these factors in the development of future therapeutics should facilitate better translational success.

## 2. Multimorbid Conditions that Influence Stroke Outcomes

**Fig. 1.**
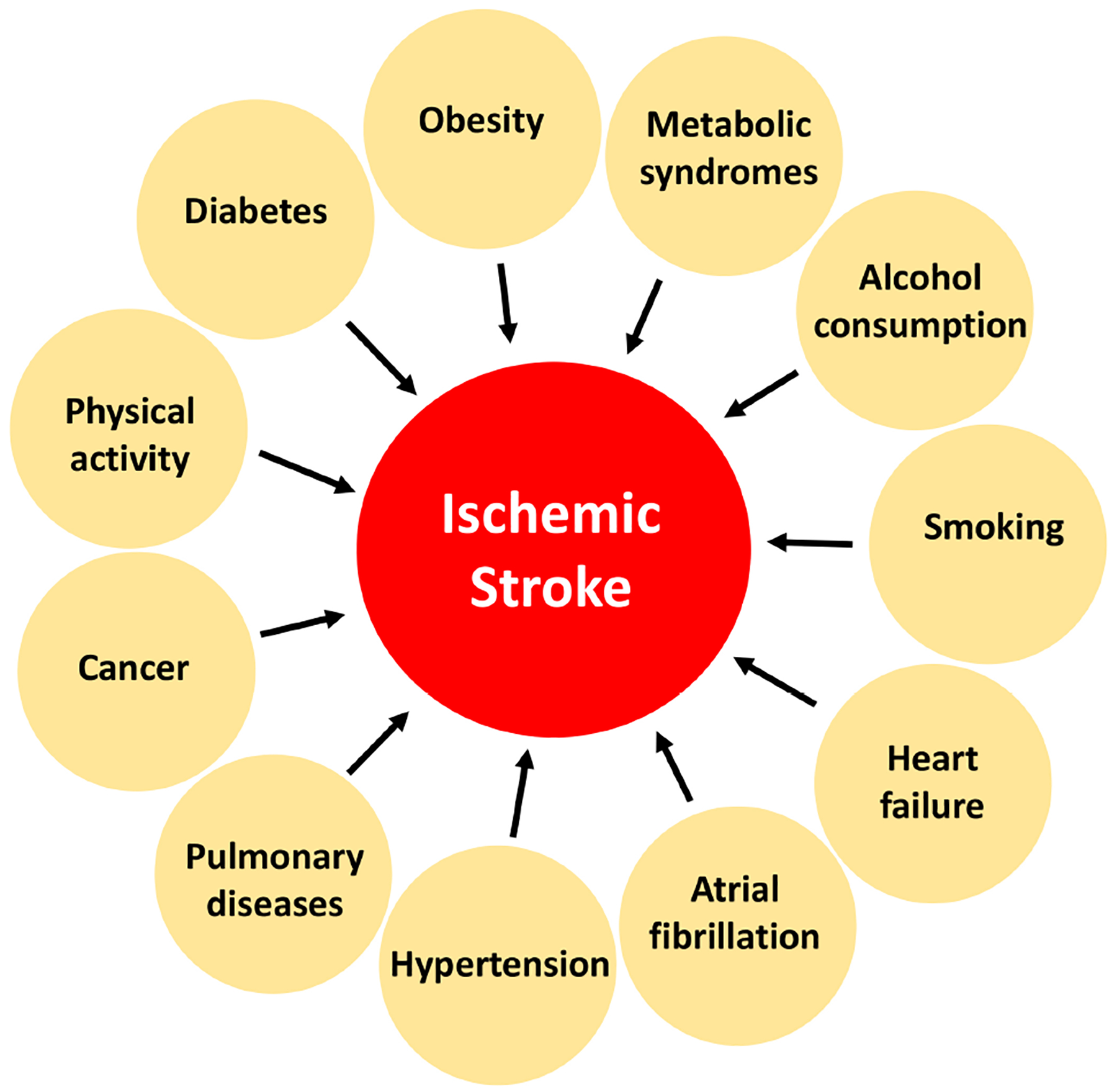
The impact of some multimorbid conditions on the outcomes of ischemic stroke.

Multimorbid conditions in stroke patients lead to a higher risk of mortality [10]. Multimorbid conditions can influence the type of treatment necessary for someone following a stroke; therefore, it is imperative to understand how these conditions influence future therapeutics for stroke patients. Diabetes, obesity, metabolic syndrome, alcohol consumption, smoking, physical activity, cancer, chronic pulmonary disease, hypertension, atrial fibrillation, and congestive heart failure are just a few multimorbid conditions seen in stroke patients [11,12] (Fig. 1). A previous study highlighted that some stroke cases are preventable, as 90% of strokes are a result of behavioral risk factors [13]. Some of these multimorbid conditions are outside the scope of this review article, and thus, will not be discussed. We will focus on the effect of cardiovascular factors on stroke outcomes, as cardiovascular conditions appear to be heavily involved in influencing the functional outcome and recovery following stroke.

According to the Center for Disease Control and Prevention, one in every six deaths from cardiovascular disease was attributed to stroke in the United States in 2018. It is well recognized that cardiac factors influence stroke outcomes. According to the Framingham study, the prevalence of stroke increased five-fold with atrial fibrillation, tripled with hypertension, and increased four-fold with heart failure [14]. Cardiovascular disease and large artery disease are responsible for up to half of the ischemic stroke cases. These cardiac diseases and risk factors are key risk factors to increase a patient’s chance of recurrent strokes [9,15]. Cardiac pathologies increase a person’s risk for stroke, underscoring that cardiac diseases are key players in stroke. The first sign of a patient experiencing a cardiac problem is usually caught during the workup following a stroke, suggesting a better diagnosis of cardiac disease is necessary to decrease the prevalence of stroke [9].

Along with cardiac factors causing a stroke, a stroke can cause cardiac disease. Previously, it was shown that patients suffering from a stroke for the first time that did not have any known cardiac diseases had a 25-fold higher chance of adverse cardiovascular events within 30 days of the stroke [16]. Following a stroke, a patient might experience a heart attack, cardiac arrest, or heart failure, leading to mortality [17]. Within 24–48 hours following a stroke, a person is at an increased risk for cardiac complications [9]. In addition to this timeframe where a person is susceptible to cardiac complications, cardiac causes of death are the second most common cause of death in acute stroke patients [18]. These findings further highlight the need for therapeutics that target the cardiovascular factors for stroke and the cardiac factors resulting from a stroke.

### 2.1 Role of Hypertension in Stroke

To date, hypertension remains the most prevalent modifiable risk factor for stroke. Blood pressure influences vascular function and organ perfusion, and cerebral circulation is highly sensitive to fluctuations in blood pressure, demonstrating the importance of regulating blood pressure to reduce the associated stroke risk [19]. Furthermore, hypertension in stroke patients increases the mortality rate, worsens functional outcomes, and increases the risk of intracranial hemorrhage following stroke [20]. There is as much as a 4-fold increase in the risk of stroke if a person has high blood pressure [21]. Studies have shown that elevated blood pressure is observed in about 75% of patients, which is associated with poorer outcomes for these individuals. Conversely, lowering blood pressure reduces the risk of stroke [22–24]. During the acute phase of ischemic stroke, blood pressure management is also beneficial for preventing hemorrhagic transformation [25]. Under the condition of reperfusion, however, higher blood pressure may be beneficial for improving cerebral blood flow in the penumbra [26]. Following a stroke, controlling blood pressure is particularly challenging, as both hypotensive and hypertensive episodes occur [27]; however, it is important to regulate blood pressure during this time to give the patient the best outcome.

Preventive options for stroke in terms of hypertension would be the maintenance of blood pressure either through lifestyle changes or maintenance medication. Improving one’s diet, exercising regularly, and quitting smoking are necessary lifestyle modifications that can decrease a person’s blood pressure and subsequently decrease a person’s risk of a stroke. Future therapeutic agents for stroke should consider their effect on stabilizing blood pressure following a stroke, as stroke patients experience a wide range of fluctuations in their blood pressure. Controlling blood pressure during this period will provide the patient with the best outcome.

### 2.2 Role of Atrial Fibrillation in Stroke

Atrial fibrillation, a preventable risk factor for stroke, increases a person’s chance of stroke five-fold [28]. In addition, atrial fibrillation accounts for more than 20% of ischemic strokes [29,30]. A stroke patient with atrial fibrillation is at high risk for recurrence, mortality, and disability [31]. The most widely accepted link between atrial fibrillation and stroke is that atrial fibrillation promotes the formation of thrombi on the interior wall of the atria. These thrombi are prone to detachment, and the detached thrombi from the left atrial wall will inevitably enter into systemic circulation and act as emboli to block distal arteries, especially small ones within end organs; hence, those blocking a small artery in the brain will cause stroke [32]. Atrial fibrillation can be easily diagnosed using an electrocardiogram (ECG), if ECG is performed on the patient during the atrial fibrillation episode. However, the impact of atrial fibrillation *per se* on heart function often is not very dramatic. Plus, not all atrial fibrillation is persistent; it can be paroxysmal or intermittent instead [33]. These make the detection of atrial fibrillation very challenging, and long-term monitoring might be necessary to detect atrial fibrillation and many other types of arrhythmia [33]. Furthermore, arrhythmia and atrial fibrillation can be a secondary event following a stroke [34].

### 2.3 Role of Congestive Heart Failure in Stroke

Congestive heart failure is another strong risk factor for stroke that increases the risk for mortality and morbidity [35]. Over 20% of patients that suffer from stroke also have heart failure [36]. Stroke patients with heart failure exhibit higher neurological deficits at admission and discharge from the hospital and this phenomenon continues even three months following the stroke [37]. Of particular interest is the efficacy of the recombinant tissue-type plasminogen activator in treating stroke patients with heart failure, as less of this therapeutic agent has been shown to be in cerebral circulation [38]. Future therapeutic agents must take this into consideration to treat stroke patients with heart failure.

### 2.4 Patent Foramen Ovale (PFO) and Ischemic Stroke

PFO is commonly associated with cryptogenic ischemic stroke, particularly in young populations [39]. Approximately 33% of strokes are cryptogenic, and these patients have a higher prevalence of PFO compared to individuals with strokes of known cause [40]. Data suggest that some cryptogenic strokes can be caused by paradoxical embolism across a PFO that can be treated medically with antithrombotic agents and percutaneously with an occluder devices. Studies from large randomized clinical trials have indicated that transcatheter PFO closure is superior to medical treatment for the prevention of recurrent stroke in young patients with cryptogenic stroke [40].

### 2.5 Role of Cardiac Misfolded Proteins in Stroke

Protein aggregates positive in ubiquitin occurs in the affected brains following transient ischemia in an ischemia/reperfusion mouse model [41,42]. Later, it was shown that reperfusion, but not ischemia, drives the formation of ubiquitin aggregates following middle cerebral artery occlusion in mice [43]. More interestingly, aggregated proteins after ischemia/reperfusion are linked to neurodegeneration diseases, such as amyotrophic lateral sclerosis and frontotemporal dementia [44], indicating the potential role of proteostasis in ischemic stroke induced brain injury. In support of this possibility, we previously demonstrated that improved proteostasis by overexpression of a ubiquitin-like protein, Ubqln1, reduces ischemic stroke-induced brain injury and enhances animal functional recovery [45,46]. Conversely, the knockout of Ubqln1 leads to the opposite results [46]. Moreover, protein aggregates are also found in peripheral organs, including the kidney, pancreas, and heart, while little is known about whether these peripherally misfolded proteins on the outcomes of the ischemic brain. A recent study from our group highlights the important effect of aberrant protein aggregation in the cardiac muscle on ischemic stroke-caused brain injury and functional recovery in mice. Specifically, mice with cardiomyocyte-restricted transgenic expression of a missense (R120G) mutant alpha B-crystallin (CryAB^R120G^) exhibit significantly increased glial activation, infarct volume, impaired functional recovery, learning and memory deficits, and increased neuroinflammation following surgically induced cerebral ischemia/reperfusion [47].

**Fig. 2.**
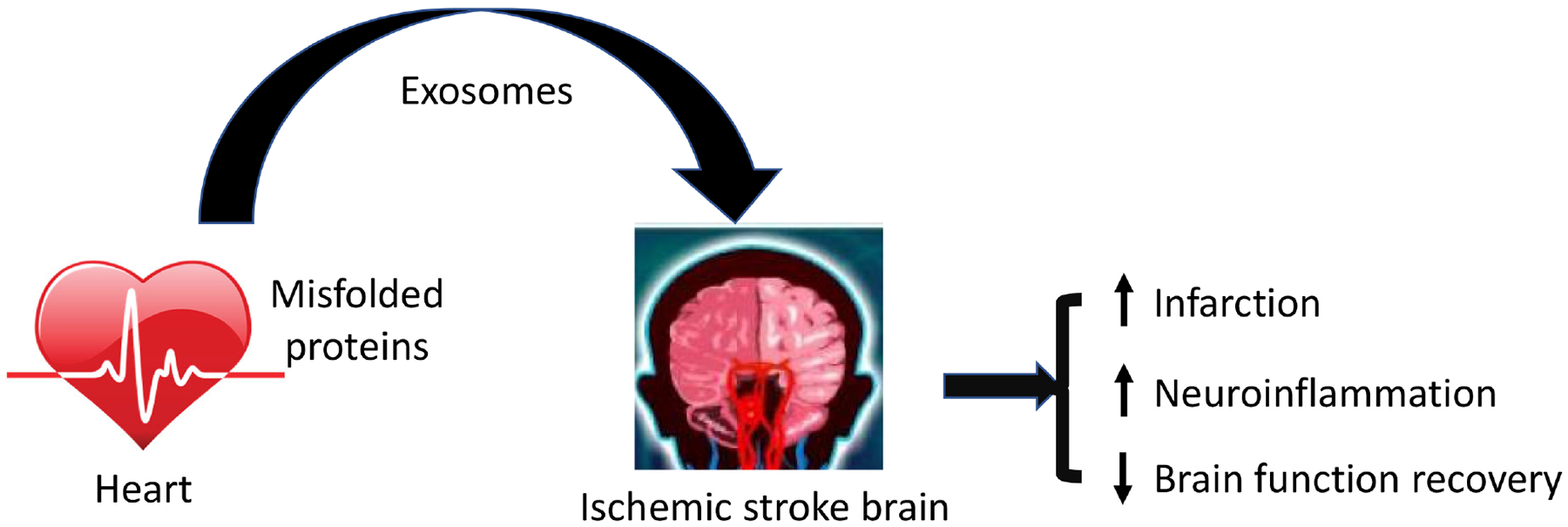
Misfolded proteins in the heart influence the outcomes of brain injury and functional recovery following ischemic stroke. Misfolded proteins are released to the bloodstream via exosomes from the cardiomyocytes and are translocated by exosomes into the brain, where they aggravate ischemic stroke induced brain injury by promoting neuronal death and neuroinflammation and impair brain functional recovery.

Although it is well known that the blood–brain barrier (BBB) is very restrictive to most proteins, the endothelial luminal membrane is, in fact, studded with specific transporters that gate the BBB and allow the selective entrance of saccharides, neutral amino acids, lipids, and vitamins as well as proteins, such as apo lipoprotein E (ApoE), insulin, and transferrin [48]. However, whether proteins from the heart could translocate to the brain and affect stroke outcomes remains unknown. The translocation of proteins from peripheral organs to the brain could occur via exosomes. Exosomes are 50–150 nanometers in diameter extracellular vesicles known for their role in intercellular communication by delivering their cargo to recipient cells [49]. As exosomes can cross the BBB, we hypothesized that exosomes could cause the propagation of misfolded proteins from the heart to the brain via the prion-like phenomenon. To test whether CryAB^R120G^ can translocate from the heart to the brain and influence functional recovery following stroke, we isolated exosomes from the plasma of CryAB^R120G^ mice and their WT littermates. While the size and concentration of exosomes did not differ between CryAB^R120G^ mice and their WT littermates, we saw a significant increase in the presence of CryAB^R120G^ and exosomal markers in the exosomes isolated from CryAB^R120G^. Moreover, following ischemia/reperfusion, plasma-derived exosomes isolated either from WT or CryAB^R120G^ mice were administered to WT mice. Mice that received plasma-derived exosomes isolated from CryAB^R120G^ mice exhibited significantly more impaired functional recovery and learning and memory impairments compared to those injected with exosomes isolated from WT mice (Fig. 2). These findings further highlight a significant role of the heart on brain functional recovery following stroke [47].

Understanding how peripheral proteins influence stroke recovery and outcomes could provide a novel mechanism to create therapeutics that target specific proteins known to influence stroke outcomes. As mentioned above, CryAB^R120G^ was translocated from the heart to the brain to influence stroke outcomes. A therapeutic agent that prevents this translocation of proteins from peripheral organs to the brain could be beneficial in improving functional outcomes and reducing the learning and memory impairments induced by stroke. Alternatively, enhanced removal of misfolded proteins either through increased ubiquitination-proteasome coupling, activation of the proteasome, or autophagy pathways in the heart should also reduce the translocation of these misfolded proteins to the brain, thus attenuating stroke-induced brain injury [45,50–52]. The therapeutic compounds previously identified to enhance the ubiquitin-proteasome system function and reduce ischemic stroke caused brain injury in mice may be used for treating stroke patients especially in the context of peripherally misfolded proteins [50,53–55].

Oxidative stress, neuroinflammation, BBB disruption, and neuronal death are the main pathophysiologies of stroke. Although protein aggregation is considered a hallmark of neurodegenerative diseases, the formation of protein aggregates can also be induced within a short time after cerebral ischemia, aggravating the cerebral ischemic injury by enhancing oxidative stress, glial activation, neuroinflammation, and neuronal death [56]. Once misfolded proteins are translocated to the brain from the heart or other peripheral organs, they interact with the normal proteins in the brain to change the normal proteins to misfolded proteins via the prion-like phenomenon [47]. Thus, protein aggregation should be considered as a stroke biomarker and represents a previously unappreciated molecular overlap between neurodegenerative diseases and ischemic stroke. However, the effect of translocation of peripheral misfolded proteins into the brain on stroke outcome in the patients remains unknown despite the limited studies in animal models. To develop more effective therapeutics for stroke patients, more studies addressing the effect of translocation of peripheral misfolded proteins are necessary in animal models, as well as in a clinical setting.

## 3. Conclusions

Cardiac risk factors are well-recognized in stroke, and a stroke can cause cardiac disease; therefore, stroke and heart disease seem to exert a double threat. As the population is aging, the risk for heart disease and stroke will only continue to rise. As a result, more people will be impacted by stroke. Any stroke patient with cardiovascular disease will suffer a worse prognosis, and treatment options for these individuals will have to be tailored to their cardiac disease. With only one FDA-approved therapeutic to treat stroke, future therapies should consider their efficacy on the cardiovascular risk factors discussed in this review, as these factors influence recovery, disability, and mortality. Future therapeutics that target not only the neurological symptoms of a stroke but also the cardiovascular factors will provide patients with a better outcome.
